# The AKT1E17K Allele Promotes Breast Cancer in Mice

**DOI:** 10.3390/cancers14112645

**Published:** 2022-05-26

**Authors:** Donatella Malanga, Carmelo Laudanna, Teresa Mirante, Fabiana Colelli, Simona Migliozzi, Pietro Zoppoli, Gianluca Santamaria, Luca Roberto, Carmela De Marco, Marzia Scarfò, Donatella Montanaro, Orlando Paciello, Serenella Papparella, Chiara Mignogna, Alfonso Baldi, Giuseppe Viglietto

**Affiliations:** 1 Department of Experimental and Clinical Medicine, University Magna Græcia of Catanzaro, 88100 Catanzaro, Italy; teresa87@tiscalinet.it (T.M.); si.migliozzi@gmail.com (S.M.); zoppolipietro@gmail.com (P.Z.); santamariagianluca@gmail.com (G.S.); cdemarco@unicz.it (C.D.M.); 2 Interdepartmental Center of Services (CIS), Department of Experimental and Clinical Medicine, University Magna Græcia of Catanzaro, 88100 Catanzaro, Italy; mignogna@unicz.it; 3 Institute for Research in Biomedicine Barcelona (IRB), 08028 Barcelona, Spain; laudanna.carmelo@gmail.com; 4 BIOGEM-Istituto di Ricerche Genetiche, 83031 Ariano Irpino, Italy; fabiana.colelli@gmail.com (F.C.); luca.roberto@biogem.it (L.R.); 5 Plaisant Polo Tecnologico s.r.l, Castel Romano, 00128 Roma, Italy; marziascrt@yahoo.it; 6 CEINGE, Biotecnologie Avanzate, 80145 Napoli, Italy; montanaro@ceige.unina.it (D.M.); alfonsobaldi@tiscali.it (A.B.); 7 Department of Veterinary Medicine and Animal Productions, Università Federico II, 80138 Napoli, Italy; orlando.paciello@unina.it (O.P.); serenella.papparella@unina.it (S.P.); 8 Department of Health Science, Università Magna Graecia, 88100 Catanzaro, Italy; 9 Department of Environmental, Biological and Pharmaceutical Sciences and Technologies, University of Campania “L. Vanvitelli”, 81100 Caserta, Italy

**Keywords:** breast cancer, AKT1E17K, transgenic mouse

## Abstract

**Simple Summary:**

The main finding reported in this manuscript is that the gain-of-function mutation AKT1E17K is a bona fide oncogene for mammary epithelium, being able to efficiently initiate breast cancer in mice. On the basis of high-molecular-weight cytokeratins expressed by AKT1E17K-derived tumors supported by additional integrative gene expression analysis these tumors resulted similar to human basal-like cancer, phenotypically and molecularly. These results indicate that the AKTE17K strain may represent an appropriate model of human basal-like breast cancer for the identification of novel therapies specific for this type of tumor.

**Abstract:**

The gain-of-function mutation in the pleckstrin homology domain of AKT1 (AKT1E17K) occurs in lung and breast cancer. Through the use of human cellular models and of a AKT1E17K transgenic Cre-inducible murine strain (R26-AKT1E17K mice), we have demonstrated that AKT1E17K is a bona fide oncogene for lung epithelial cells. However, the role of AKT1E17K in breast cancer remains to be determined. Here, we report the generation and the characterization of a MMTV-CRE; R26-AKT1E17K mouse strain that expresses the mutant AKT1E17K allele in the mammary epithelium. We observed that AKT1E17K stimulates the development of mammary tumors classified as ductal adenocarcinoma of medium–high grade and presented a variety of proliferative alterations classified as adenosis with low-to-high grade dysplasia in the mammary epithelium. A subsequent immunohistochemical characterization suggested they were PR^−^/HER2^−^/ER^+^, basal-like and CK8^−^/CK10^−^/CK5^+^/CK14^+^. We also observed that, in parallel with an increased proliferation rate, tumors expressing mutant AKT1E17K presented an activation of the GSK3/cyclin D1 pathway in the mammary epithelium and cluster significantly with the human basal-like tumors. In conclusion, we demonstrate AKT1E17K is a bona fide oncogene that can initiate tumors at high efficiency in murine mammary epithelium in vivo.

## 1. Introduction

AKT, identified as the human homolog of the v-akt murine thymoma viral oncogene belongs to a small family of three protein kinases (AKT1, AKT2, and AKT3) related to protein kinase A, G, and C. Hence, it is also denoted as protein kinase B (PKB) [1].

AKT kinases represent the primary downstream endpoint of the phosphoinositide 3-kinase (PI3K) signaling pathway, regulating proliferation, survival, metabolism, and invasion [2]. Active AKT exerts its functions through the phosphorylation of a wide range of substrates including the glycogen synthase kinase 3 beta, GSK3-β, the cell cycle inhibitors p27 and p21, the pro-apoptotic protein BAD, and the RHEB GTPase [3]. Notably, aberrant activation of the PI3K–AKT pathway is involved in the development and/or progression of several tumor types, including breast cancer [4].

Breast cancer is the most diagnosed cancer in women (30% of expected 175,000 female cancer cases in Italy) (www.aiom.it; accessed on 15 October 2020). So far, little progress has been made to improve overall survival of breast cancer patients, with only targeted therapy directed against tumors overexpressing HER2 being able to significantly improve patients’ outcome [5]. Recent genomic studies on the breast cancer landscape have shown that breast cancer displays numerous genomic alterations in actionable oncogenes [6], including PIK3CA and AKT [7]. In fact, aberrant signaling through the PI3K/AKT pathway, detected as increased AKT phosphorylation, occurs in approximately 70% of cases, and has been associated with poor prognosis, tumor recurrence and resistance to therapy [8,9].

In mammals, there are three AKT isoforms encoded by three different genes (AKT1/PKBα, AKT2/PKBβ, and AKT3/PKBγ). AKT isoforms are differentially expressed both temporally and spatially: AKT1 and AKT2 are ubiquitous and regulate cell size and metabolism, whereas AKT3 expression is mainly restricted to brain and testes and regulates brain size and architecture [10].

Hyperactivation of the PI3K–AKT pathway is involved with progression in the majority of tumor types [4,11], though the role of each AKT isoform remains unclear, despite the fact that each isoform may appear amplified or mutated in different cancer types. In breast cancer, AKT activation is apparently an early event, since phosphorylated AKT is observed in ductal carcinoma in situ [12] and AKT1 mutations are detected at a high frequency in benign papilloma [13]. The most frequent AKT1 mutation results from the replacement of glutamic acid with lysine at residue 17 (AKT1E17K) and it has been identified in several types of solid tumors [14,15,16,17]. The mutant AKT1E17K protein shows increased affinity for PI(4,5)P2, which allows for its recruitment to plasma membrane and constitutive activation [15,18]. The detected frequency of AKT1E17K mutation in breast cancer ranges between 1.4% and 8.2%, with a mean mutation frequency of 3.8%, and is apparently restricted to estrogen receptor (ER)-positive tumors [13,16].

The AKT1E17K allele has transforming capacity in fibroblasts in vitro and induces leukemia in mice [15,19], but inconclusive information and discordant results have been provided.

The aim of this work was to address the role of AKT1E17K in the transformation of breast epithelial cells in vivo. We demonstrate that AKT1E17K is a bona fide oncogene that is able to initiate cancer in the mammary gland at high efficiency. Importantly, AKT1E17K-driven tumors are ER-positive and, phenotypically and molecularly, similar to human basal-like cancer. 

## 2. Materials and Methods

### 2.1. Generation and Analysis of Transgenic Mice

The generation of R26-AKT1E17K transgenic mice has been previously described [20]. R26-AKT1E17K mice were mated with MMTV-Cre mice (strain B6129-Tg MMTV-Cre1Mam/J, #003551; Jackson Laboratory) to generate R26-AKT1E17K; MMTV-Cre. MMTV-Cre mice are frequently used to generate mouse models of human breast cancer because the mouse mammary tumor virus LTR promoter directs the expression of Cre recombinase, and there are high levels of recombination in the virgin and lactating mammary gland driving transgene expression in the mammary gland tissue in a conditional Cre-lox mouse model. Five 6–8-week-old breeding pairs of were used to establish the colony, following a breeding scheme for mouse models that incorporate the Cre/lox system. (https://www.jax.org/news-and-insights/2011/september/most-efficient-breeding-scheme-for-generating-cre-lox-tissue-specific-or-in; accessed on 23 September 2011). R26 wild-type; MMTV-Cre (indicated as R26MMTV-Cre) littermates were used as controls in all experiments. R26 wild-type or non-expressing Cre were not included in the study. Same sex (female) mutant and control mice were used in all experiments. Animal experimentation was approved by the local ethical committee “Comitato Etico per la Sperimentazione Animale” (CESA) of Biogem (Ariano Irpino, Italy) and conformed to the regulations and guidelines of Italy and the European Union. All efforts were made to minimize animal suffering (ARRIVE release 2.0 Checklist in Supplemental Section). Mice were housed in specific-pathogen-free (SPF) conditions and maintained in IVC cages under constant conditions of temperature (22 ± 2°C), humidity (55% ± 10 UR) and 12 h cycles of light and dark. Mice had free access to irradiated standard diet and water. The mice were sacrificed by euthanasia with carbon dioxide and mammary glands were collected. Mammary tissue and tumors were divided into 2 halves: one half was snap-frozen, the other half was fixed in formalin or mechanically dissociated in single cell suspension. Mice were genotyped by PCR of tail DNA [21]. Genotypes of R26-AKT1E17K; MMTV-Cre mice were determined using the primers listed as follows: *AKT1E17K* primers: 5′ARMF, 5′-AACTGCAGACTTGTGGGATAC-3′; 3′ARMR, 5′-ATATTAGTCCACCTCACTCCT-3′; mE17KR5 5′-GCCAACCCTCCTTCACAATA-3′; primers for the deletion of loxP-flanked transcriptional stop sequences:

TRF, 5′-GGATCGACGGTATCGTAGAGTCGAGGCCG-3′
L2R 5′-GCCAATGAAGGTGCCATCATTCTTGAGGAGGAAG-3′; primers for MMTV-Cre: MMTV-F, 5′-CTGATCTGAGCTCTGAGTG-3′
MMTV-R 5′-GTGAAACAGCATTGCTGTCACTT-3′.

### 2.2. Western Blot and Antibodies

Whole-tissue protein extracts were prepared with NP-40 buffer (10 mM Tris–HCl pH 7.5, 150 mM NaCl, 1% NP-40) containing protease inhibitors (SigmaFast, Sigma-Aldrich). Western blot analysis was carried out by standard methods [14]. Anti-phospho-AKT (#4058), anti-AKT1 (#2938), anti-phospho-GSK3-α/β (#9331), anti-GSK3-α/β (#9338), and Cyclin D1 (#2978) were purchased from Cell Signaling Technology (Danver, MA, USA). 

### 2.3. Quantitative Reverse Transcription Real-Time PCR (qRT-PCR)

Total RNA was prepared as described [22]. RT-PCR was performed on RNA extracted by Trizol (Invitrogen) and retro-transcribed with SuperScript II (Invitrogen). RT-PCR was performed using the Power SYBR Green PCR Master Mix in ABI Prism 7900 thermocycler (Applied Biosystems, Foster City, CA). The relative amounts of mRNA were calculated by the comparative cycle threshold (CT) method [23]. 

### 2.4. Enzymatic Dissociation of Murine Mammary Glands

Mammary tissues were mechanically dissociated into small pieces using a surgical blade. Biopsies were placed into digestion medium (DMEM/F12) (Life Technologies, Foster City, CA, USA) supplemented with 200 U/mL collagenase (Sigma-Aldrich) and 100 U/mL Hyaluronidase (Sigma-Aldrich, St. Louis, MO, USA) for 5 h at 37 °C. After enzymatic digestion, cell suspensions were filtered through 100, 70, 40, and 20 µm meshes (BD, San Jose, CA, USA) and centrifuged at 1200 rpm for 5 min. After PBS washing, pellets were resuspended in 5 mL of RBC buffer (155 mM NH_4_Cl; 10 mM KHCO_3_ or NaHCO_3_; 0.1 mM EDTA) to eliminate erythrocytes. After PBS washing, pellets were resuspended, and cells were counted using a staining solution made with 1 volume of cell suspensions diluted 1:1 with 0.4% Trypan Blue solution (Sigma-Aldrich). 

### 2.5. Histological Analysis and Immunohistochemistry

Mammary glands and tumors were collected, fixed with 10% formalin and embedded in paraffin using standard procedures. Sections (5 μm) were mounted onto slides and stained with hematoxylin and eosin to be evaluated by pathologists. Antibodies used for immunostaining were: anti-ER-α (sc-542, Santa Cruz, CA, USA), anti-HER2 (#4290, Cell Signaling Technology), anti-PGR (NCL-L-PGR-312, Dako, CA, USA), anti-Cytokeratin 5 (PRB-160P, Covance, Princeton, NJ, USA), anti-Cytokeratin 8 (10R-C177AX, Fitzgerald Industries International, Acton, MA, USA), anti-Cytokeratin 14 (E2624, Panomics, Fremont, CA, USA), and anti-Cytokeratin 10 (PRB-159P, Covance). Immunostaining was performed with standard protocols using Bond™ Polymer Refine Detection (Leica Biosystem, Buffalo Grove, IL, USA) according to the manufacturer’s instructions.

### 2.6. Gene Expression Profiling

RNA concentration and quality were determined with Agilent TapeStation 2200 (Agilent Technologies, Santa Clara, CA, USA). For each sample, 500 ng of total RNA were used to synthesize biotinylated cRNA with Illumina RNA Amplification Kit (Ambion, Austin, TX, USA). Synthesis was carried out according to the manufacturers’ instructions. cRNA concentration and quality were assessed by Agilent TapeStation 2200 (Agilent Technologies). Technical replicates were produced and 750 ng cRNA were hybridized for 18h to MouseWG-6 v2.0 Expression BeadChip (Illumina, San Diego, CA, USA). Hybridized chips were washed and stained with streptavidin conjugated Cy3 (GE Healthcare, Milan, Italy). BeadChips were dried and scanned with an Illumina iScan (Illumina). Gene expression analysis was performed using Gene-Spring 13.1 (Agilent Technologies, Santa Clara, CA, USA). Multichip average (RMA) normalization and principal component analysis (PCA) were performed using Gene-Spring 13.1 (Agilent Technologies, Santa Clara, CA, USA). Fold-change threshold was set at ≥1.5 and statistical significance was set at *p* ≤ 0.05 by *t*-test. The Array data are made publicly available upon publication of the paper at https://www.ebi.ac.uk/arrayexpress/experiments/E-MTAB-7388/, accession number: E-MTAB-7388.

### 2.7. GSEA Analysis

To perform GSEA analysis, we first selected genes that were differentially expressed in 4 human breast cancer subtypes: luminal A, luminal B, basal, and HER2-enriched on the basis of previous studies [24,25]. Subsequently, the selected human signatures were converted to murine signatures using the Ensembl GRCm38.p6 database. Finally, the ranked gene lists were generated by multiplying the log10(*p*-value) and the Fold-change sign to determine the *p*-value [26]. The gene set used for GSEA analysis was “Canonical pathways and experimental signatures curated from publications” (C2). Genes were grouped according to “Gene ontology” (GO) and “Hallmark gene sets” (H) from the Molecular Signature Database (MSigDB) [27]. Hierarchical clustering of the leading-edge genes was performed as described by Ward and Jaccard [28]. The Hallmark classes denoted “Late Estrogen” and “Early Estrogen” were merged into one denoted “Estrogen Response”.

### 2.8. Integrative Analysis of Murine Models of Breast Cancer

The transcriptomic profiles resulting from 365 breast tumor samples derived from 27 different genetically modified murine models reported in the Gene Expression Omnibus (GSE3165, GSE8516, GSE10450, GSE14753, GSE15263, GSE17916, GSE20645, GSE22150, GSE27101, GSE30866, GSE31942, GSE32152, GSE34479, and GSE42640) [29,30] were independently downloaded and RMA normalization was applied [31]. Data integration was performed by the “InSilicoMerging” R package 1.14.0. Only the probesets present in all the 365 GEO datasets and in the profiles of tumors deriving from R26-AKT1E17K; MMTV-Cre mice (6591 genes) were considered. Classification analysis was performed using “mixOmics” R package 6.1.3. Briefly, we used a supervised version of partial least squares (PLS) regression [32] called sparse PLS-Discriminant Analysis (PLS-DA) [33,34,35]. Quantile normalization was applied to achieve a common distribution of intensities between samples and genes. 

### 2.9. Validation of Murine Gene Expression Data in the Human Breast Cancer Dataset from TCGA

The human breast cancer dataset from The Cancer Genome Atlas (TCGA) collection (BRCA) was used. The gene expression dataset derived from 519 breast tumor samples profiled with Agilent chip G4502A and 521 samples profiled with RNA-seq. Raw data obtained from Agilent gene expression were scaled with quantile normalization. The level 3 RNA-Seq data were downloaded from the TCGA by the R TCGA bio links package [36]. GC correction and full quantile were applied, according to [37]. The hierarchical clustering algorithm based on the Ward linkage method and Euclidean distance was performed on cluster samples and genes [38].

### 2.10. Statistical Analysis

Quantitative (q)RT-PCR data are expressed as means ± SD of at least three independent experiments conducted in triplicates. Statistical significance was evaluated by *t*-tests, as indicated in Figure legends. Statistical significance in the Kaplan–Meyer curves was assayed by the log rank (Mantel–Cox) test. For the planning of experimental size, Statistical Power Analyses by G*Power was used, with the following parameters: power 0.8; α error 0.05, and effect size 0.07, considering the possible biological effect by the review of the published data relative to other AKT transgenic mouse models. Statistical significance was indicated as follows: *p* ≤ 0.05 (*), *p* ≤ 0.01 (**), *p* ≤ 0.001 (***) and *p* ≤ 0.0001 (****).

## 3. Results

### 3.1. Mutant AKT1E17K Promotes Mammary Tumor Development

We have recently generated a Cre-inducible knock-in mouse strain that allows for the expression of the mutant AKT1E17K allele after the deletion of the lox-P flanked transcriptional stop sequence [20]. In this study, we have addressed the functional significance of AKT1E17K in breast cancer. To this aim, we have crossed R26AKT1E17K mice with a MMTV-Cre strain that expresses the P1 Cre recombinase under the control of the MMTV-LTR promoter. The resulting R26AKT1E17K; MMTV-Cre mice express the mutant AKT1E17K allele in both virgin and lactating mammary glands at high efficiency. 

Immunoblotting analysis with anti-AKT1 and anti-phosphoAKT1 Ser473 antibodies demonstrated the expression of the mutant AKT1E17K allele in the whole mammary gland of R26AKT1E17K; MMTV-Cre mice. The expression of the transgene induced an increased mammary gland cellularity (Supplemental Figure S1). 

**Figure 1:**
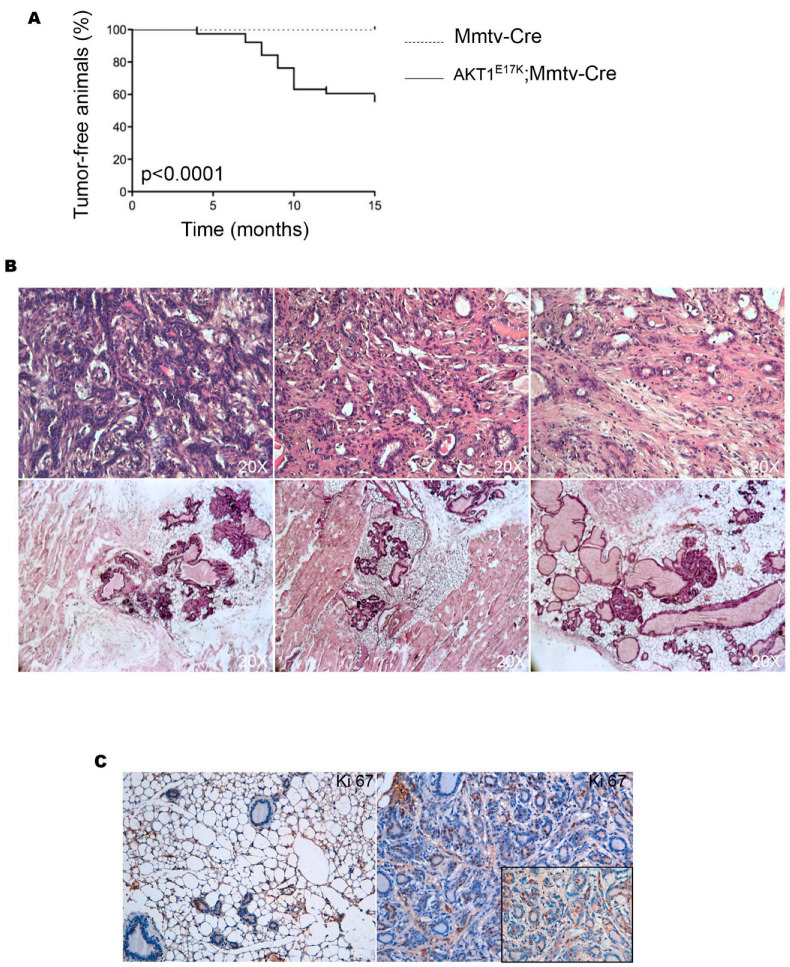
Histologic analysis of tumors derived from R26AKT1E17K; MMTV-Cre mice. (**A**) Kaplan–Meier curves estimating the percentage of tumor-free mice (R26AKT1E17K; MMTV-Cre mice, n = 35; MMTV-Cre mice, n = 35); (**B**) representative staining with hematoxylin and eosin of mammary glands explanted from R26AKT1E17K; MMTV-Cre mice; (**C**) left panel, representative immunostaining of Ki67 in normal mammary gland; right panel, representative immunostaining of Ki67 in breast cancer derived from R26-AKT1E17K; MMTV-Cre mice. Magnification: 20×. Magnification in insets: 40×.

We next investigated whether the expression of AKT1E17K in the breast epithelium is sufficient to promote the development of breast cancer in vivo. Two cohorts of R26AKT1E17K; MMTV-Cre mice and R26-MMTV-Cre littermates were monitored weekly for evidence of palpable tumors for 15 months. R26AKT1E17K; MMTV-Cre mice developed palpable tumors starting from 4 months of age, both in virgin and multiparous mice. The kinetics of the spontaneous tumor development observed in a cohort of 35 R26AKT1E17K; MMTV-Cre mice and 35 control mice are reported in Figure 1A. The incidence of tumors in R26AKT1E17K; MMTV-Cre mice was about 43%. Conversely, no control mouse developed palpable tumors during the observation period. 

Histological analysis of mammary glands explanted from R26AKT1E17K; MMTV-Cre mice confirmed the presence of tumors classified as ductal adenocarcinomas to medium or high grade (Figure 1B, upper panels). Furthermore, R26AKT1E17K; MMTV-Cre mice presented a variety of proliferative alterations as adenosis with low- or high-grade dysplasia (Figure 1B, lower panels). The Ki67 staining revealed that the mean number of positive nuclei in the mammary glands of R26AKT1E17K; MMTV-Cre mice (n = 5) was on average 40%, whereas normal mammary glands of control mice were almost negative for Ki67 staining (almost <2% positive cells), see Figure 1C.

### 3.2. Characterization of Mammary Tumors Developed in R26AKT1E17K; MMTV-Cre Mice

**Figure 2:**
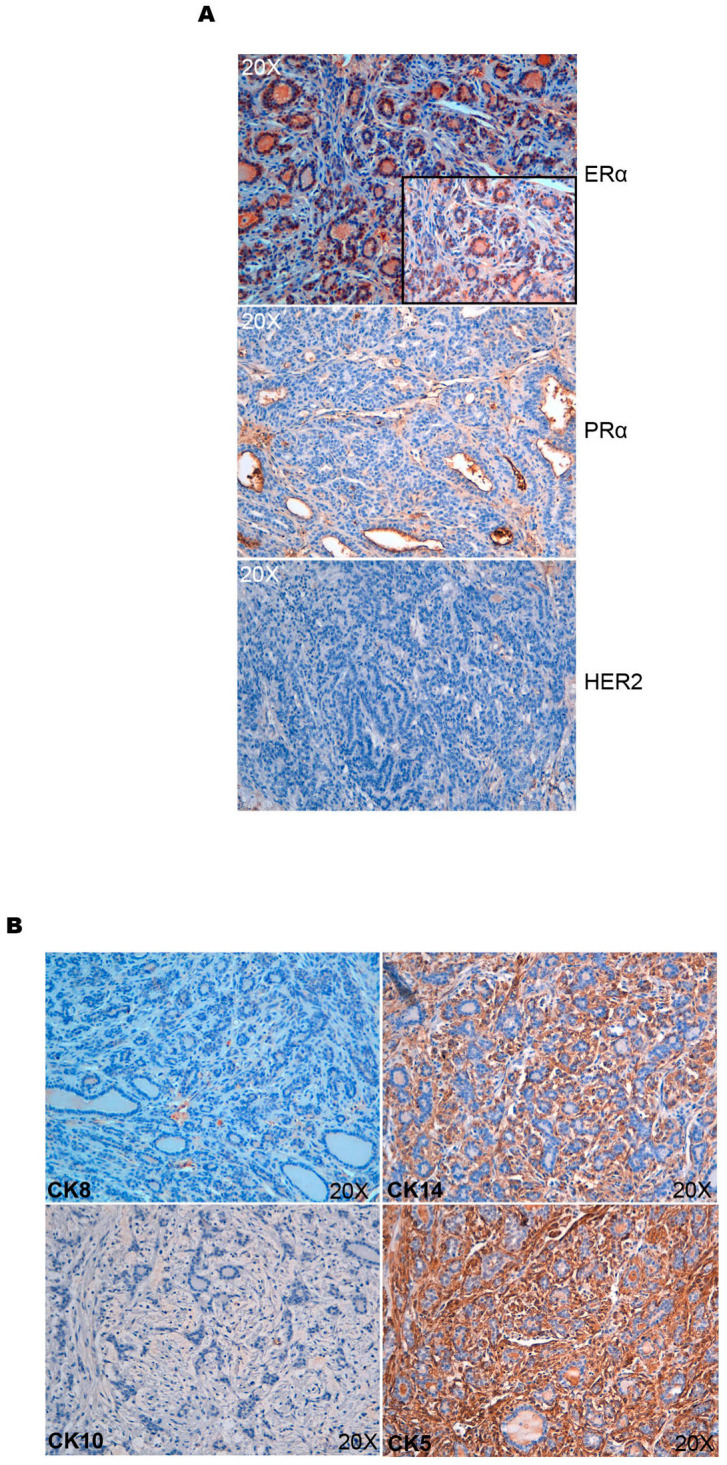
Characterization of tumors derived from R26AKT1E17K; MMTV-Cre mice (**A**) representative immunostaining of ERα, PRα, and HER2 performed on tumors explanted from R26AKT1E17K; MMTV-Cre mice; (**B**) representative immunostaining of CK8, CK14, CK10, and CK5 in tumors explanted from R26AKT1E17K; MMTV-Cre mice. Magnification: 20×. Magnification in insets: 40×.

In order to characterize the tumors induced by AKT1E17K in mice, we performed expression analysis for ERα, PR, and HER2. Tumors explanted from R26AKT1E17K; MMTV-Cre mice were positive for ERα, negative for PR and HER2, and thus, could be classified as ER^+^/PR^−^/HER2^−^, see Figure 2A. However, medium-grade tumors presented a higher level of ER compared with high-grade tumors, which were still considered expressing ER.

Immunostaining for CK5, CK8, CK10, and CK14 showed that mammary tumors induced by AKT1E17K had positive results for CK5 and CK14 and negative for CK8 and CK10, suggesting that tumors developed in R26AKT1E17K; MMTV-Cre mice are basal-like, as reported in Figure 2B. 

### 3.3. Mutant AKT1E17K Promotes Activation of AKT-Pathway in Mouse Mammary Tumors

We investigated whether the expression of AKT1E17K in mammary epithelium of R26AKT1E17K; MMTV-Cre mice resulted in increased phosphorylation of AKT1 and in the activation of the GSK3/cyclin D1 pathway. Considering this aim, we studied the phosphorylation status of AKT1 and of its substrate GSK3 using phospho-specific antibodies (Supplemental Figure S2). We observed that tumors explanted from R26AKT1E17K; MMTV-Cre mice presented increased levels of pAKT1 at S473 and pGSK at S9/22 in comparison with the corresponding normal mammary gland; tumor tissues also exhibited elevated levels of cyclin D1 (Supplemental Figure S2). The expression level of PTEN resulted normal (data not shown).

Overall, these results indicated that active AKT1E17K expressed in the murine mammary epithelium is able to activate GSK3α/β, leading to increased cyclin D1 levels, which in turn contributes to the observed increased proliferation.

### 3.4. Gene Expression Analysis of Breast Tumors in R26AKT1E17K; MMTV-Cre Mice

To characterize mammary tumors induced by AKT1E17K, we performed microarray-based gene expression analysis of mRNAs extracted from tumors and normal mammary glands explanted from R26AKT1E17K; MMTV-Cre. In particular, we profiled tumors derived from R26AKT1E17K; MMTV-Cre classified as poorly differentiated (n = 3) and moderately differentiated (n = 2). As control, we used gene expression profiles of normal mammary glands explanted from R26AKT1E17K; MMTV-Cre that did not present palpable tumors (n = 4) and normal mammary glands derived from R26MMTV-Cre littermates (n = 3). 

**Figure 3:**
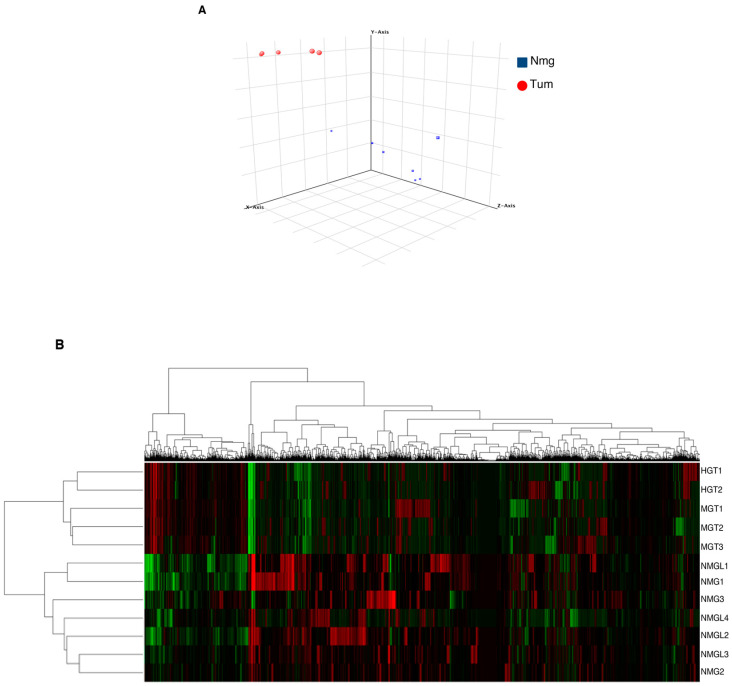
Gene expression analysis of mammary glands explanted from R26AKT1E17K; MMTV-Cre mice. (**A**) 3D principal component analysis (PCA) plot of the expression profiles derived from tumors (T = 5, red) of R26AKT1E17K; MMTV-Cre mice and normal mammary tissue (N = 7, blue). (**B**) Gene set clustering analysis of NMG and tumors.

The MouseWG-6 v2.0 Expression BeadChip containing 45,200 mouse transcripts was used to obtain transcriptional profiles of tumors and normal glands. The profiles of both tumors and normal glands were homogeneous, as shown by PCA in Figure 3A and the unsupervised cluster analysis in Figure 3B, respectively. 

For the rest of the manuscript, the normal mammary tissue used as the control (N) refers to normal mammary glands from both mice. Gene profiles were compared by Gene Set Enrichment Analysis (GSEA), using Hallmark gene sets by mSigDB. The analysis of gene profiles revealed that cell cycle and cell proliferation categories were over-represented in tumor profiles, with cell-cycle-associated genes highly expressed in tumors induced by AKT1E17K. The top-scoring Hallmark gene sets enriched in tumors were “E2F targets” enriched in genes encoding cell cycle-related targets of the E2F family of transcription factors, “G2M Checkpoint”, enriched in genes of the G2/M checkpoint, and “MYC targets V1” that consisted of genes whose transcription was directly regulated by MYC (Supplemental Table S1). The complete list of Hallmark gene sets is reported in Supplemental File S1. Real-time PCR validation of representative differentially expressed genes is reported in Supplemental Figure S3.

### 3.5. Molecular Classification of Tumors Induced by AKT1E17K in Mice

Subsequently, we compared, at the molecular levels, mammary tumors induced by mutant AKT1E17K with those promoted by other oncogenes (i.e., p53, BRCA1, PIK3CA, Myc, Neu). Considering this, we made use of an integrative approach implemented in the mixOmics R-package [39] that combines raw data derived from independent gene expression experiments conducted in 27 murine models of breast cancer (see Materials and Methods for the Gene Expression Omnibus datasets), divided into three subtypes: luminal (n = 8), basal (n = 17), and claudin-low (n = 2). 

**Figure 4:**
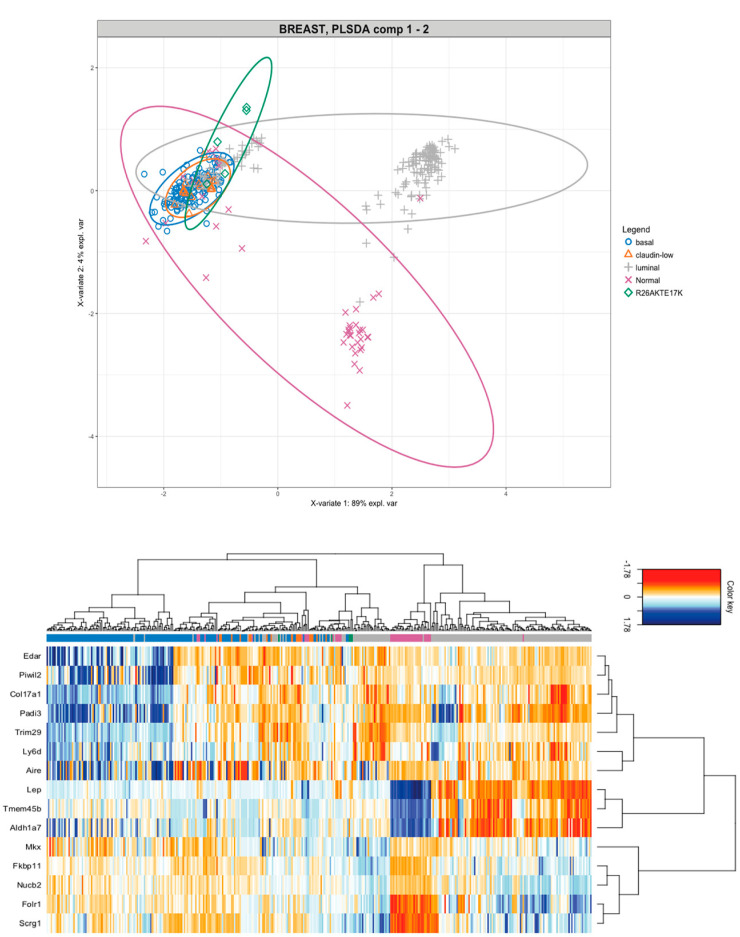
Comparison of tumors induced by mutant AKT1E17K in mice with pre-existing murine models of breast cancer. Upper panel SPLS-DA representation. Confidence ellipses for each class have been plotted to highlight the strength of discrimination (CI 95%). SPLS-DA transformation ensures that the Principal Component 1 (PC1) on the horizontal axis has the highest variation (variability > 89%) and the PC2 on the vertical axis has the second highest variation (>4%). Lower panel Heatmap representing the gene signature derived from PC1, PC2, and PC3 analyses. Arrows indicate the tumors derived from R26AKT1E17K; MMTV-Cre mice.

The resulting dataset comprises 365 samples divided into 148 basal tumors, 157 luminal tumors, 18 claudin-low tumors, and 42 normal samples [29,30]. A supervised analysis allowed the identification of the lowest number of components that were able to discriminate among the three subtypes. In agreement with the results of immunohistochemistry, mammary tumors from R26AKT1E17K; MMTV-Cre mice clustered within the basal-like subgroup of breast cancer (i.e., MMTV-Met, FVB-C3-Tag, MMTV-Wnt, PI3KCA, DMBA-induced) [40,41,42,43]—see the ellipse plot in Figure 4 upper panel for a representation of components 1 and 2 of the analysis (>95% of the variance; *p*-value < 0.05), whereas the ellipse plot that represents components 1 and 3 is reported in Supplemental Figure S4.

We also identified the most informative genes that discriminated among the three subtypes: five genes in the component 1 (Car6, Acox2, Scrg1, Folr1, Mkx), three genes in the component 2 (Tmem45b, Art3, Amy1), seven genes in the component 3 (Piwil2, Aire, Edar, Padi3, Col17a1, Apobec1, and Il23a) and 98 genes in the component 4, See Figure 4 lower panel. The specific contribution of the genes to each component is reported in Supplemental Figures S5–S8. 

**Figure 5:**
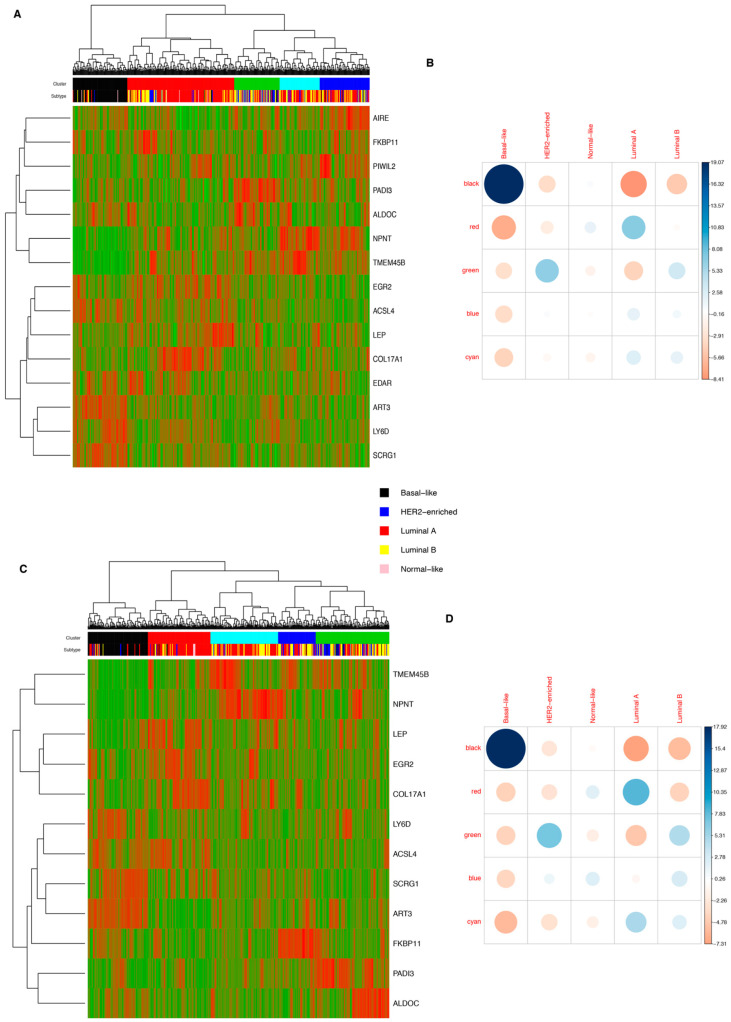
Unsupervised hierarchical cluster analysis of TCGA dataset by using “15 murine gene signature”. (**A**) Clustering of 504 Array-profiled breast cancer samples derived from TCGA. In the heatmap, rows correspond to genes and columns to samples grouped, applying a hierarchical clustering of the expression matrix. The degree of gene expression is represented in the scale bar; (**B**) Pearson’s chi-squared test relative to the enrichment of the molecular subtypes of breast cancer of Array-profiled samples. Balloon plots represent the difference between the observed and expected sample number. Balloon colors indicate statistical significance: blue indicates positive values; red indicates negative values. Balloon size indicates the strength of association between clusters and tumor subtypes; (**C**) clustering of 521 RNA-seq-profiled breast cancer samples derived from TCGA. In the heatmap, rows correspond to genes and columns to samples grouped, applying a hierarchical clustering of the expression matrix. The degree of gene expression is represented in the scale bar; (**D**) Pearson’s chi-squared test relative to the enrichment of the molecular subtypes of breast cancer of RNA-seq-profiled samples. Balloon plots represent the difference between the observed and expected sample number. Balloon colors indicate statistical significance: blue indicates positive values; red indicates negative values. Balloon size indicates the strength of association between clusters and tumor subtypes.

Finally, we investigated whether the gene signature identified in the integrative analysis of murine models was useful for the classification of human breast cancer. Considering this, we selected the 15 most informative genes (Car6, Acox2, Scrg1, Folr1, Mkx, Tmem45b, Art3, Amy1, Piwil2, Aire, Edar, Padi3, Col17a1, Apobec1, IL-23a) identified by an analysis of murine models, and performed an unsupervised hierarchical clustering analysis, making use of two different human gene datasets contained in the TCGA (i.e., Array gene expression dataset comprising 504 patients, RNAseq gene expression dataset comprising 521 patients), as shown in the heatmaps of Figure 5A,C. A chi-squared test was performed to determine the significance of the enrichment (*p*-value < 2 × 10^−16^). 

In particular, this murine 15-gene signature led to the enrichment of almost all basal samples in one “basal-like” cluster, both in the Array gene expression dataset (82/94 of total basal tumor samples) and in the RNAseq dataset (83/96 of total basal tumor samples)—see Figure 5B,D for statistical analysis. It is noteworthy that in the RNAseq analysis, the PIWIL2, EDAR, and AIRE genes were not significant because they did not pass the quality of the applied filters.

### 3.6. Comparison of the Expression Profile of Tumors Induced by AKT1E17K in Mice with the Profile of Human Breast Cancer

Human breast cancer is classified according to tumor type (ductal or lobular infiltrating carcinoma), histological grade (I–III), steroid hormone receptor status (positive or negative), and HER2 status (positive or negative). In addition, gene profiling of human breast cancer has allowed the definition of at least five additional subgroups, including luminal A (LumA), luminal B (LumB), human epidermal growth factor receptor 2 (HER2)-enriched, basal-like, and normal-like [24,25,44]. 

**Figure 6:**
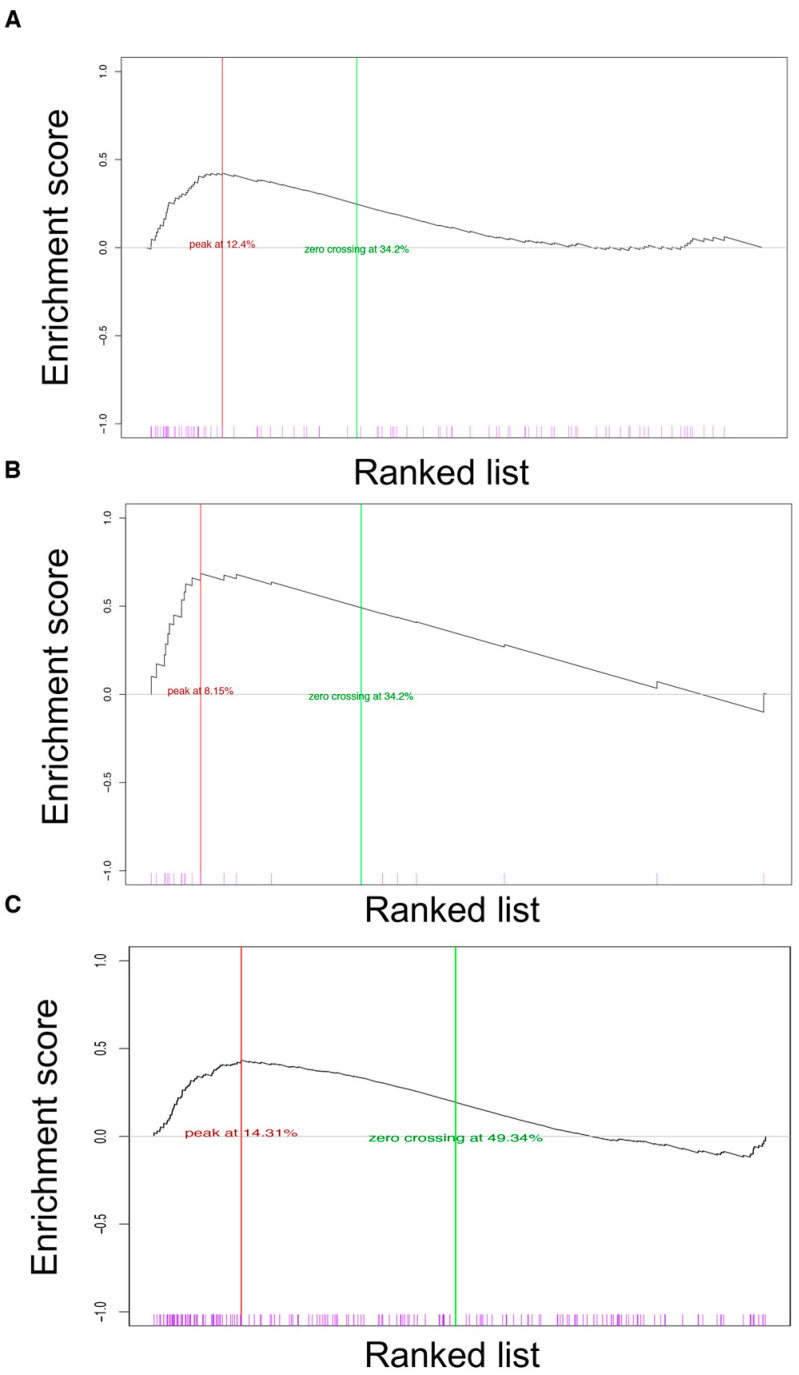
GSEA of tumors derived from R26AKT1E17K; MMTV-Cre mice. (**A**) GSEA plot of the enrichment score (ES) relative to the comparison between profiles of mammary tumors derived from R26AKT1E17K; MMTV-Cre mice and human basal/luminal A subtypes. The maximum ES is indicated; (**B**) GSEA plot of ES relative to the comparison between profiles of mammary tumors derived from R26AKT1E17K; MMTV-Cre mice and human basal/luminal B subtypes. The maximum ES is indicated; (**C**) GSEA plot of ES relative to the comparison between profiles of mammary tumors derived from R26AKT1E17K; MMTV-Cre mice and the “Estrogen receptor” Hallmark gene set collections obtained by the mSigDB of human tumors.

Therefore, we compared gene expression profiles of tumors induced by mutant AKT1E17K in mice with the profiles derived from human breast cancer present in public datasets [24,25]. First, four different human gene signatures made of differentially expressed genes enriched in four different human cancer subtypes (luminal A, luminal B, basal-like, and HER2+) were generated, as described in Materials and Methods—see also Supplemental File S2. Second, the human genes contained in the four lists (luminal A, luminal B, basal-like, and HER2+) were converted into the murine orthologues by Ensembl database annotation (EMBL-EBI). Third, we generated a ranked murine gene list (mRL) containing the murine genes that were differentially expressed in the breast tumors derived from R26AKT1E17K; MMTV-Cre mice (*t*-test, *p*-value < 0.05)—see Supplemental File S3. Fourth, we tested the obtained mRL with the human signatures denoted luminal A, luminal B, basal-like and HER+ by GSEA. This analysis showed that the tumors promoted by AKT1E17K in the mouse markedly resemble the basal-like subtype of human breast cancer—see Figure 6A,B.

Subsequently, we compared the murine mRL with a human ranked gene list (hRL) containing genes enriched in the human basal-like subtype by GSEA [25]. A significant overlap was obtained by the evaluation of GO and C2 categories. We found that 16 out of the 23 Hallmark gene sets that were significant by mSigDB in human basal-like breast cancer were also enriched in the tumors derived from R26AKT1E17K; MMTV-Cre mice, as reported in Table 1.

In total, 6 out of 10 GO and 51 out of 100 canonical pathways C2, resulting from the profiles of AKT1E17K tumors, were significantly enriched in the human gene expression data, as reported in Supplemental Table S2 and Supplemental File S4.

Notably, the Hallmarks denoted as E2F targets, G2M checkpoint, MYC targets, and Mitotic spindle were the most significantly enriched in both mice and humans. Conversely, the estrogen responsive early and estrogen responsive late pathways, which results showed they were significantly enriched by GSEA in AKT1E17K tumors, were not enriched in human basal-like breast cancer, see Figure 6C. The complete list of Hallmarks significantly enriched in mouse and human basal-like tumors is reported in Supplemental File S5.

## 4. Discussion

In this study, we present experimental evidence that AKT1E17K is a bona fide oncogene for mammary epithelium, being able to efficiently initiate breast cancer in mice by using a ROSA26-based engineered mouse model for the expression of the oncogenic AKT1(E17K) mutation in the mammary gland.

In human breast cancer, a somatic AKT1E17K mutant has been observed with a mean mutation frequency of 3.8% (range 1.4–8.2%). Notably, this mutation is associated with ER expression and is observed in carcinoma in situ and benign papilloma [12,13], suggesting that it represents an early event in mammary tumorigenesis [13,45]. This observation is in line with the notion that aberrant AKT activation represents an early event in breast cancer development associated with poor prognosis, tumor recurrence, and resistance to therapies [46].

Several genetically modified mouse strains have been generated to model the role of the PI3K/AKT pathway in breast cancer, including mammary-specific knockout of PTEN [47], mammary-specific expression of mutant PIK3CA, or myristoylated AKT1 [48].

The main finding reported in this manuscript is that the gain-of-function mutation AKT1E17K is a bona fide oncogene for mammary epithelium. We report that this specific AKT1 mutant is able to initiate mammary tumors in female mice at high efficiency. Transgenic AKT1E17K mice also presented a variety of proliferative alterations of the mammary epithelium that were classified as adenosis with low-to-high grade dysplasia.

These results are in partial contrast to a previous study which showed that the transgenic expression of an AKTE17K allele in mouse breast epithelium induces gland hyperplasia, but is not sufficient to induce mammary tumors [49,50]. Additionally, other constitutively active AKT1 mutants (AKT1-308D-473D, myr-AKT1) apparently induce pre-neoplastic lesions and have been shown to require additional events, such as a loss of Tp53 or chemical carcinogens to progress to fully malignant tumors [48,51]. However, at the same time, active AKT1 is necessary for mammary tumor development driven by ErbB2 or PyMT, since its ablation in double transgenic mice interferes with tumor growth [52,53].

This discrepancy may be related to the level of AKT activation in the mammary gland of transgenic mice. On one hand, the expression of the transgene may not be sufficient to initiate cancer. On the other hand, the level and/or the activity of the oncogenic transgene may be so high that it activates differentiation mechanisms that protect mammary epithelium from tumor development. The observation that enforced AKT activation, following PTEN loss or myr-AKT expression, leads to p53- or p27-dependent senescence checkpoints, suggesting that this may be the case [52,54,55].

This situation is reminiscent of what is observed in human cell lines. Initially, the adoptive expression of AKT1E17K in immortalized breast MCF-10A cells was shown to exert minimal effects [56], but subsequent studies have shown that AKT1E17K is indeed able to stimulate cell proliferation, migration, and chemoresistance [57].

Notably, tumors that originated in the mammary epithelium of ROSA26-AKT1 mice are ductal adenocarcinoma of medium-to-high grade. Immunohistochemistry analysis demonstrated that these tumors were ER+/PR- and HER2-. Such an observation is in line with previous studies that have suggested the existence of a functional link between AKT and ER. Signaling through the PI3K/AKT pathway modulates ERa activity and AKT mediates signaling from the membrane to the nuclear ERa [58]. Moreover, AKT activity is associated with antiestrogen resistance in human breast cancer cell lines and affects Erα DNA binding properties. Finally, cancers that develop in MMTV-AKT1 mice upon exposure to chemical carcinogens are Erα-positive [53] and AKT1E17K mutations are confined to ER-positive ductal and appear to be restricted to ductal and lobular histotypes [16,45]. 

High-molecular-weight cytokeratins, such as CK5 and CK14, are expressed in the basal cells of the mammary epithelium and are considered basal cytokeratins; whereas CK8 and CK10 are considered luminal cytokeratins. Tumors derived from AKT1E17K mice were positive for the expression of CK5 and CK14 and negative for CK8 and CK10, suggesting that these tumors resemble the human basal-like subtype. The basal-like phenotype of the tumors caused by the expression of AKT1E17K is also supported by additional integrative gene expression analysis.

In humans, basal tumors account for 70% to 80% of cases classified as triple-negative [59]. These tumors are of particular interest for their aggressiveness and lack of any systemic therapy. The results of the gene expression analysis from the mammary tumors caused by AKTE17K performed here were compared with the transcriptomes of mammary tumors that originated in several murine models of breast cancer including Trp53, c-Myc, and SV40 LT. This analysis reveals that tumors derived from AKTE17K mice cluster only with the sample derived from basal-like mouse models.

Notably, we have found that the transcriptomes of murine AKTE17K-dependent tumors cluster significantly with the transcriptomes of human basal-like tumors, high expression of basal markers, high expression of proliferation-related genes, and activation of the GSK3/cyclin D1 pathway. Moreover, we confirmed the expression of some of the genes such as PIWIL4, Col17a1, LY6D, and EGR2 in the AKTE17K-dependent tumors, genes that were previously described to be expressed in the human tumors classified as basal-like/triple-negative [42,60].

These results indicate that the AKTE17K strain may represent an appropriate model of human basal and/or triple-negative breast cancer for the development of novel therapies for these patients. 

## 5. Conclusions

Several genetic studies suggest that the activation of the PI3K/AKT pathway contributes to BC tumorigenesis, and the major human BC subgroups showed the activation of this specific signaling pathway. Our work represents the first mouse model that developed mammary tumors dependent on the constitutively activation of AKT1 by E17K substitution. Furthermore, using genomic analysis, we can identify the unique gene set in an AKT1E17K mouse model that reflects the “basal like” phenotype in humans. These results emphasize the relevance of this model for studying this specific subgroup of human cancer BC. A more comprehensive examination of the genomic features of the tumors revealed a specific gene expression profiling and pathway enrichment, providing a more complete framework of the different aspects of breast cancer biology.

## Figures and Tables

**Table 1:** Table of selected gene sets from Hallmark MsigDB collection enriched in both tumors derived from R26AKT1E17K; MMTV-Cre mice and human BC “basal-like”.

	R26AKT1E17K; MMTV-Cre			Human BC “Basal-Like”		
Hallmark Gene Set	es	*p*-Value	NES	es	*p*-Value	NES
E2F TARGETS	0.64	0.00	2.64	0.76	0.00	2.53
G2M CHECKPOINT	0.59	0.00	2.42	0.73	0.00	2.42
MYC TARGETS V1	0.57	0.00	2.30	0.67	0.00	2.20
MITOTIC SPINDLE	0.51	0.00	2.06	0.53	0.00	1.78
MTORC1 SIGNALING	0.32	0.02	1.30	0.48	0.00	1.59
WNT BETA CATENIN SIGNALING	0.52	0.00	1.67	0.52	0.03	1.40
TNFA SIGNALING VIA NFKB	0.39	0.00	1.60	0.38	0.03	1.24
INFLAMMATORY RESPONSE	0.33	0.01	1.35	0.41	0.01	1.35
MYC TARGETS V2	0.44	0.01	1.48	0.68	0.00	1.97
GLYCOLYSIS	0.36	0.00	1.46	0.39	0.02	1.28
UNFOLDED PROTEIN RESPONSE	0.47	0.00	1.79	0.42	0.02	1.33
PEROXISOME	0.41	0.00	−1.57	−0.44	0.02	−1.38
FATTY ACID METABOLISM	0.53	0.00	−2.11	−0.44	0.01	−1.42
BILE ACID METABOLISM	0.43	0.00	−1.65	−0.50	0.00	−1.54
ESTROGEN RESPONSE LATE	0.43	0.00	1.79	−0.56	0.00	−1.85
ESTROGEN RESPONSE EARLY	0.36	0.00	1.48	−0.69	0.00	−2.28

## Data Availability

Array data available at https://www.ebi.ac.uk/arrayexpress/experiments/E-MTAB-7388/, accession number: E-MTAB-7388 upon publication of the paper.

## Supplementary Materials

The following supporting information can be downloaded at: https://www.mdpi.com/article/10.3390/cancers14112645/s1, Figure S1: Generation of transgenic R26AKT1E17K; MMTV-Cre mouse. Figure S2: Western blot analysis of Akt pathway. Figure S3: Real-time PCR validation. Figure S4: SPLS-DA representation of principal components 1–3. Figures S5–S8: Classification analysis “mixOmics” 6.1.3 in the component 1–4. Table S1: Table of selected gene sets from Hallmark MsigDB collection enriched in the tumors from R26AKT1E17K; MMTV-Cre mice. Table S2: Table of selected gene sets from GO mSigDB collection enriched in the tumors derived from R26AKT1E17K; MMTV-Cre mice and TCGA BC dataset. Supplemental Files S1–S5: Gene Set Enrichment Analysis (GSEA): “Canonical pathways and experimental signatures curated from publications” (C2). “Gene ontology” (GO) and “Hallmark gene sets” (H) from the Molecular Signature Database (MSigDB).
