# Supplementary material for: The AKT1E17K Allele Promotes Breast Cancer in Mice
Source: Cancers (Basel). 2022 May 26;14(11):2645. doi: 10.3390/cancers14112645 (PMC9179273; doi:10.3390/cancers14112645)
Supplement: Supplementary file 1 [file cancers-14-02645-s001.zip › Supplementary Figures & Tables.pdf]

Table S1. Table of selected gene sets from Hallmark MsigDB collection enriched in the tumors from R26AKT1E17K;MMTV-Cre mice.

Gene set	es	p-value	NES
E2F TARGETS	0,64	0	2,64
G2M CHECKPOINT	0,59	0	2,42
MYC TARGETS V1	0,57	0	2,30
MITOTIC SPINDLE	0,51	0	2,06
EPITHELIAL-MESENCHYMAL TRANSITION	0,50	0	2,05
P53 PATHWAY	0,49	0	2,02
DNA REPAIR	0,50	0	1,98
PROTEIN SECRETION	0,51	0	1,88
UNFOLDED PROTEIN RESPONSE	0,47	5,0E-04	1,79
ESTROGEN RESPONSE_LATE	0,43	0	1,79
WNT/β-CATENIN SIGNALING	0,52	0,0015	1,67
PI3K/AKT/MTOR SIGNALING	0,44	0	1,66
ANDROGEN RESPONSE	0,44	5,0E-04	1,65
TNF-α SIGNALING VIA NFKB	0,39	5,0E-04	1,60
APICAL JUNCTION	0,38	5,0E-04	1,57
IL6/JAK/STAT3 SIGNALING	0,43	0,0035	1,57
APOPTOSIS	0,38	5,0E-04	1,55
TGF-β SIGNALING	0,46	0,0055	1,52
ESTROGENRESPONSE EARLY	0,36	0,0015	1,48
MYC TARGETS V2	0,44	0,009	1,48

Table S2. Table of selected gene sets from GO mSigDB collection enriched in the tumors derived from R26AKT1E17K; MMTV-Cre mice and TCGA BC dataset.

	R26AKT1^{E17K};MMTV-Cre			TCGA		
**GO Gene set**	**Rank**	**es**	**NES**	**Rank**	**es**	**NES**
NEGATIVE REGULATION OF RETINOIC ACID RECEPTOR	1	0,90	2,62	NV	NV	NV
**KINETOCHORE**	**2**	**0,68**	**2,57**	**8,00**	**0,69**	**2,16**
**SISTER CHROMATID SEGREGATION**	**3**	**0,64**	**2,56**	**1,00**	**0,70**	**2,26**
**CHROMOSOME CENTROMERIC REGION**	**4**	**0,64**	**2,56**	**5,00**	**0,68**	**2,20**
**SISTER CHROMATID COHESION**	**5**	**0,67**	**2,51**	**3,00**	**0,72**	**2,25**
**CONDENSED CHROMOSOME CENTROMERIC REGION**	**6**	**0,69**	**2,50**	**2,00**	**0,73**	**2,25**
REGULATION OF RETINOIC ACID RECEPTOR SIGNALING PATHWAY	7	0,79	2,37	NV	NV	NV
**MITOTIC SISTER CHROMATID SEGREGATION**	**8**	**0,64**	**2,34**	**6,00**	**0,72**	**2,17**
RETINOIC ACID RECEPTOR BINDING	9	0,67	2,22	NV	NV	NV
DNA REPLICATION INDEPENDENT NUCLEOSOME ASSEMBLY	10	0,68	2,15	NV	NV	NV

A

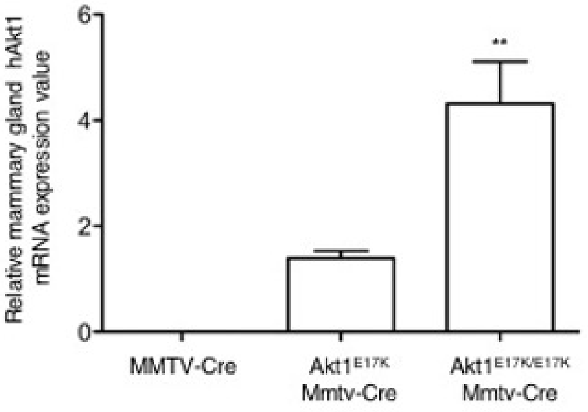

B

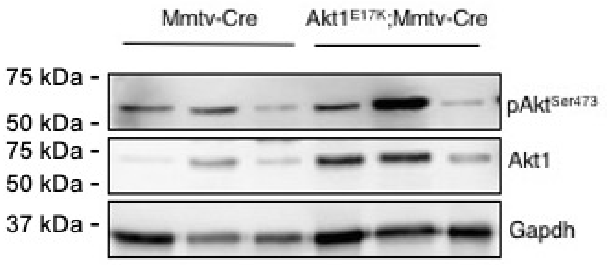

C

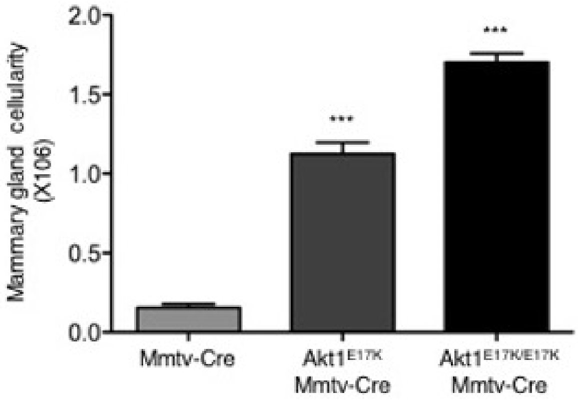

Figure S1. Generation of transgenic R26AKT1E17K; MMTV-Cre mouse. (**A**) Relative mRNA expression of human AKT1E17K allele by qRT-PCR in mammary tissues explanted from homozygous (n=8) and heterozygous (n=8) R26AKT1E17K; MMTV-Cre mice or control R26; MMTV-Cre littermates (n=7). Data indicate the mean ± SD from at least triplicated experiments. **p=0.0029. (**B**) Representative immunoblot analysis of pAKT and total AKT1 in protein extracts from whole mammary glands derived from R26AKT1E17K;MMTV-Cre and MMTV-Cre littermates.

(**C**) Trypan blue analysis of cell viability in single cell suspensions from enzymatic dissociation of whole mammary glands explanted from R26AKT1E17K;MMTV-Cre (n=5) and MMTV-Cre litterma-tes (n=5), respectively. Data indicate mean ± SD from at least triplicated experiments.

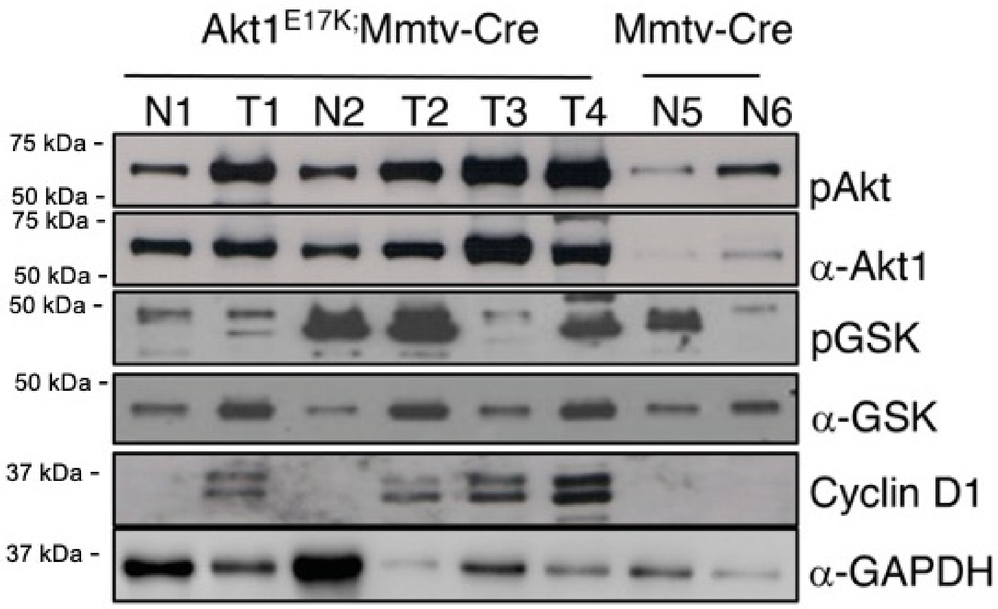

Figure S2. Immunoblot analysis of S473-phosphorylated AKT1 (pAKT), total AKT1, S9/22-phos-phorylated GSK3α/β (pGSK3), total GSK3α/β (GSK3), Cyclin D1 and GAPDH performed in protein extracts derived from normal mammary tissues or tumors explanted from R26AKT1^{E17K};MMTV-Cre mice or in normal mammary tissues derived from MMTV-Cre mice.

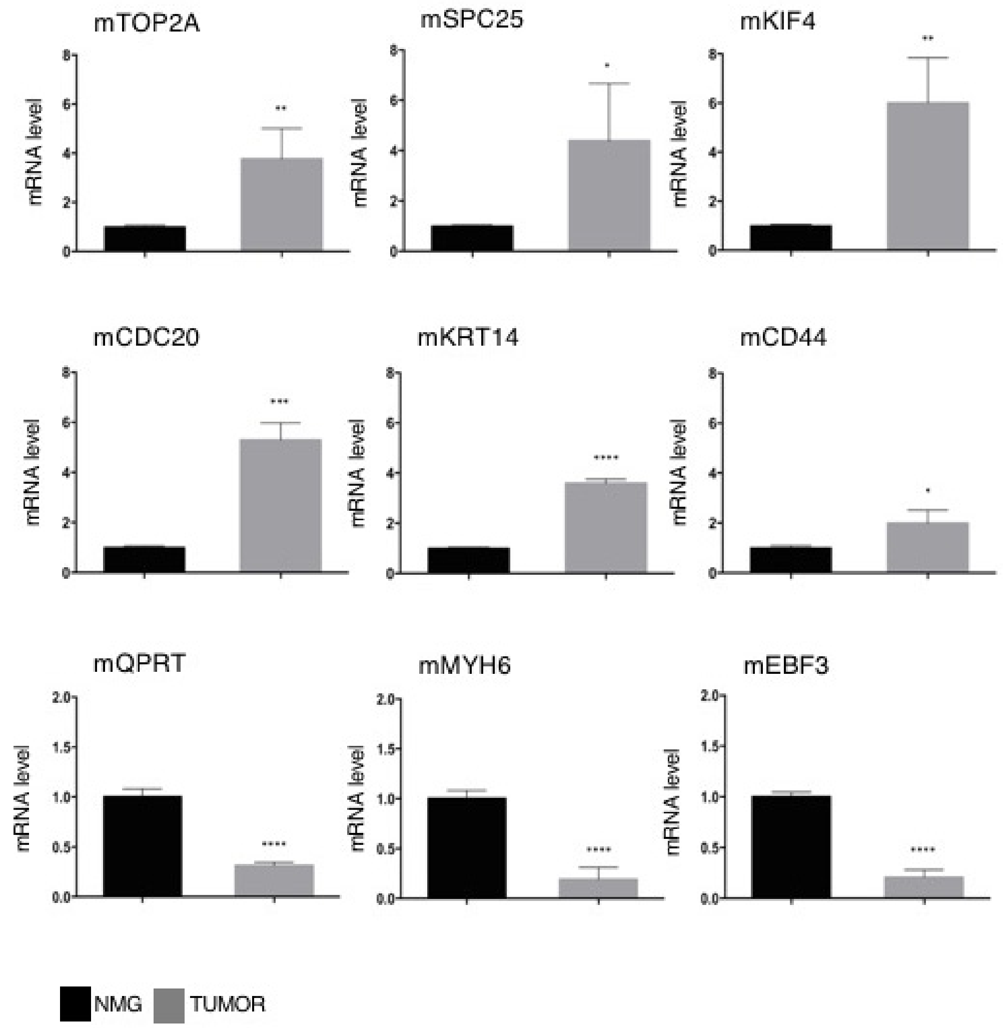

Figure S3. Real Time PCR validation of representative differential expressed genes. The relative amounts of mRNA were calculated by the comparative cycle threshold (CT) method (23). Quantitative (q)RT-PCR data are expressed as means ± SD of at least three independent experiments conducted in triplicates. Statistical significance was evaluated by t-tests as indicated.

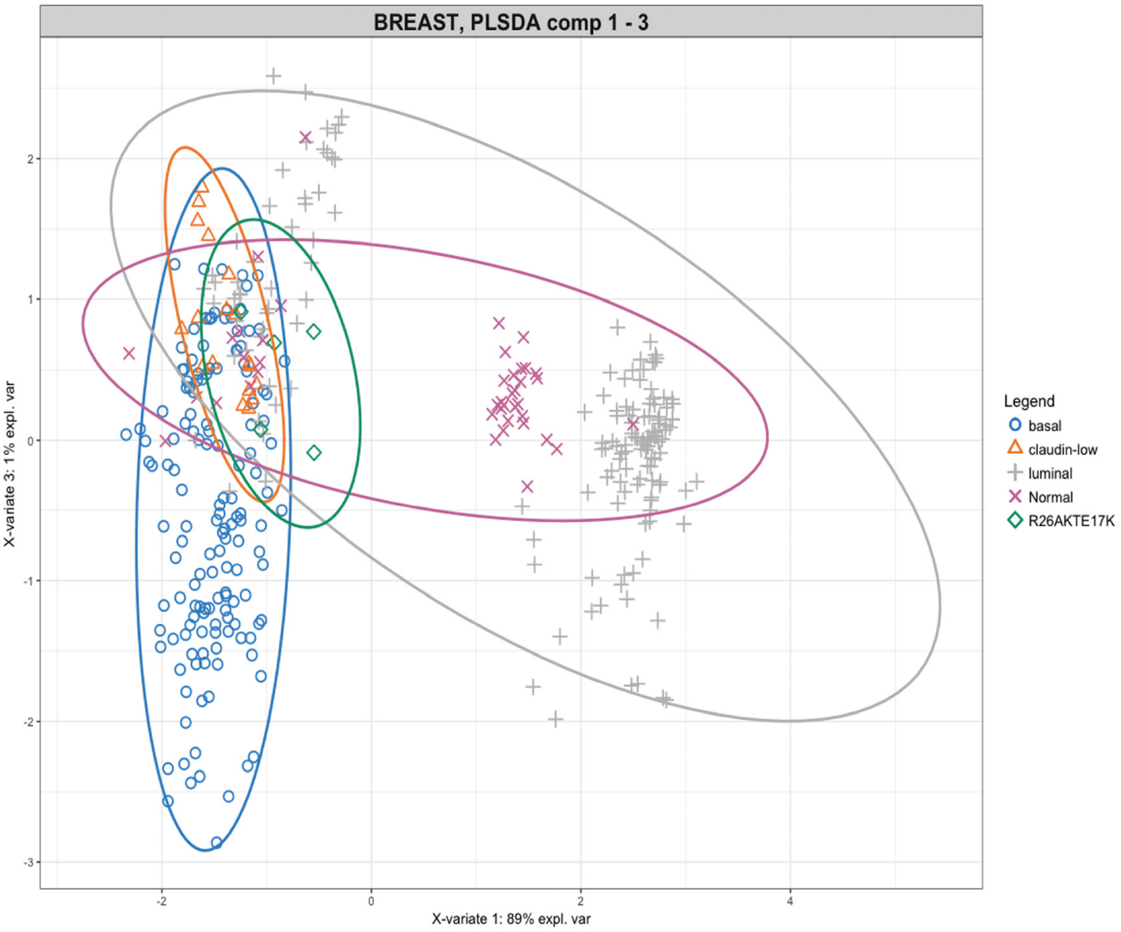

Figure S4. SPLS-DA representation of Principal Component 1 (PC1) on the horizontal axis has the highest variation (variability >89%) and the PC3 on the vertical axis has the second highest variation (>1%), p-value<0.05.

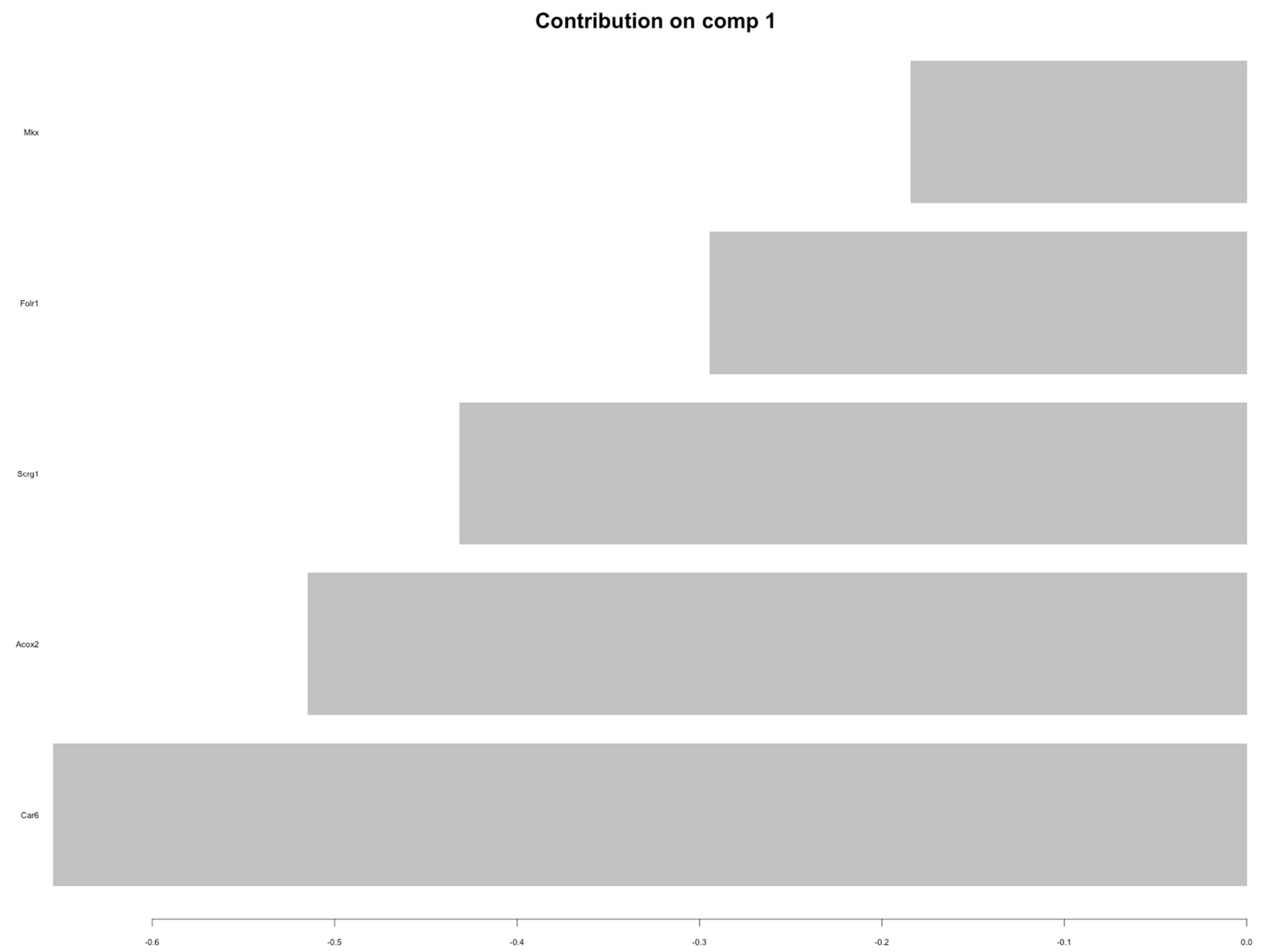

Figure S5. Classification analysis was performed using “mixOmics” R package 6.1.3. In the figure are reported the genes that discriminated among the three subtypes, luminal (n=8), basal (n=17) and claudin-low (n=2) in the component 1.

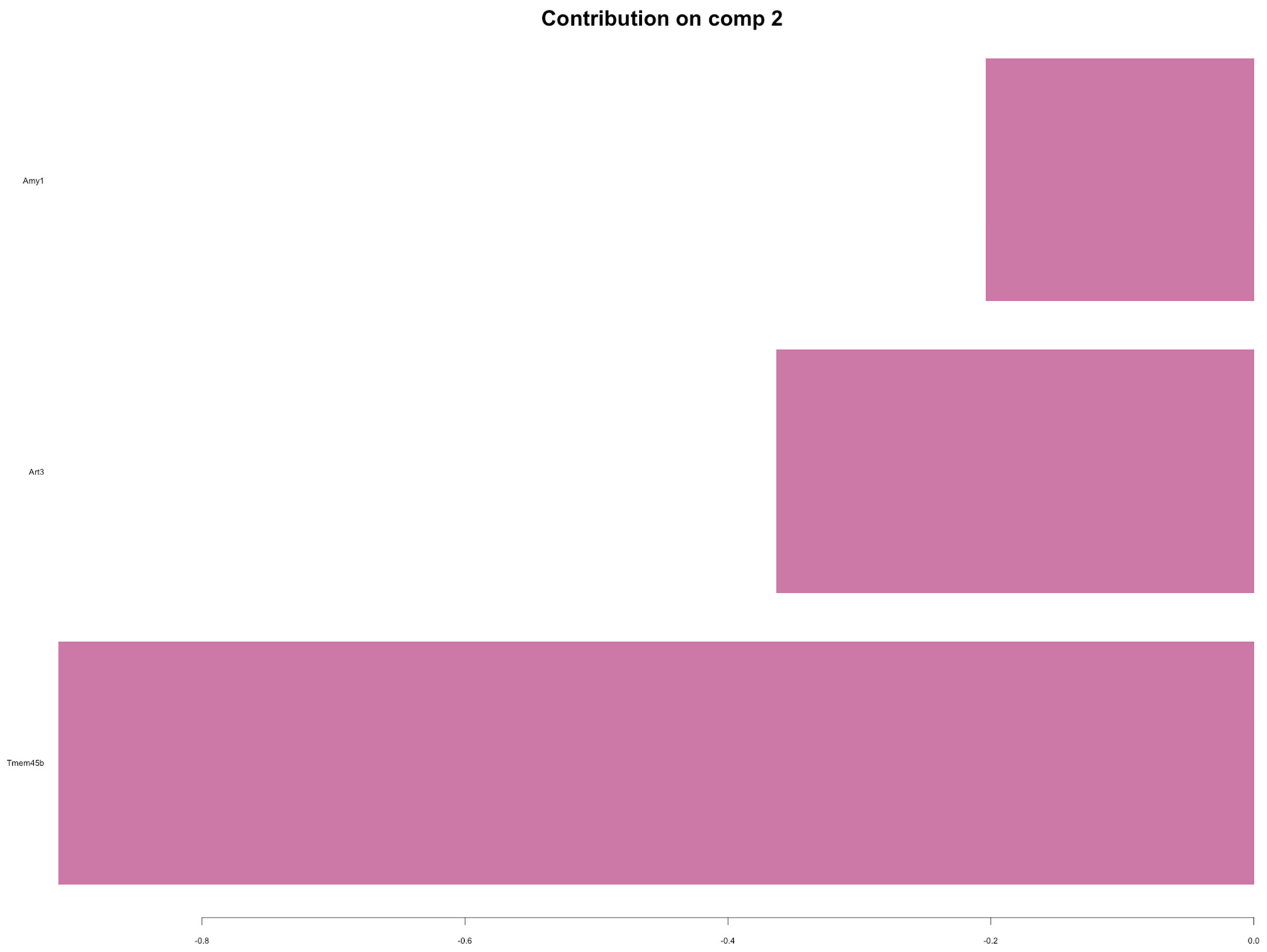

Figure S6. Classification analysis was performed using "mixOmics" R package 6.1.3. In the figure are reported the genes that discriminated among the three subtypes, luminal (n=8), basal (n=17) and claudin-low (n=2) in the component 2.

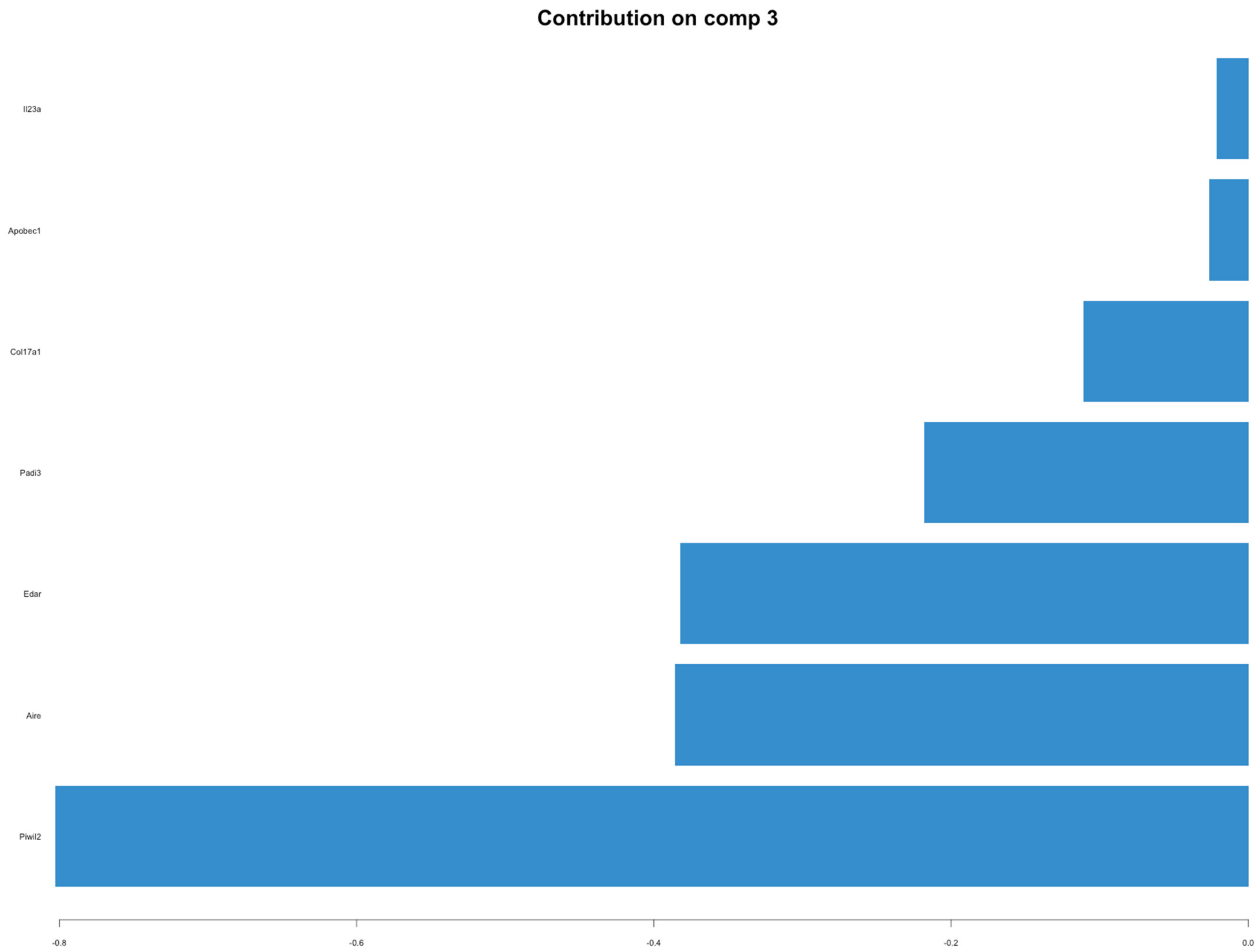

Figure S7. Classification analysis was performed using "mixOmics" R package 6.1.3. In the figure are reported the genes that discriminated among the three subtypes, luminal (n=8), basal (n=17) and claudin-low (n=2) in the component 3.

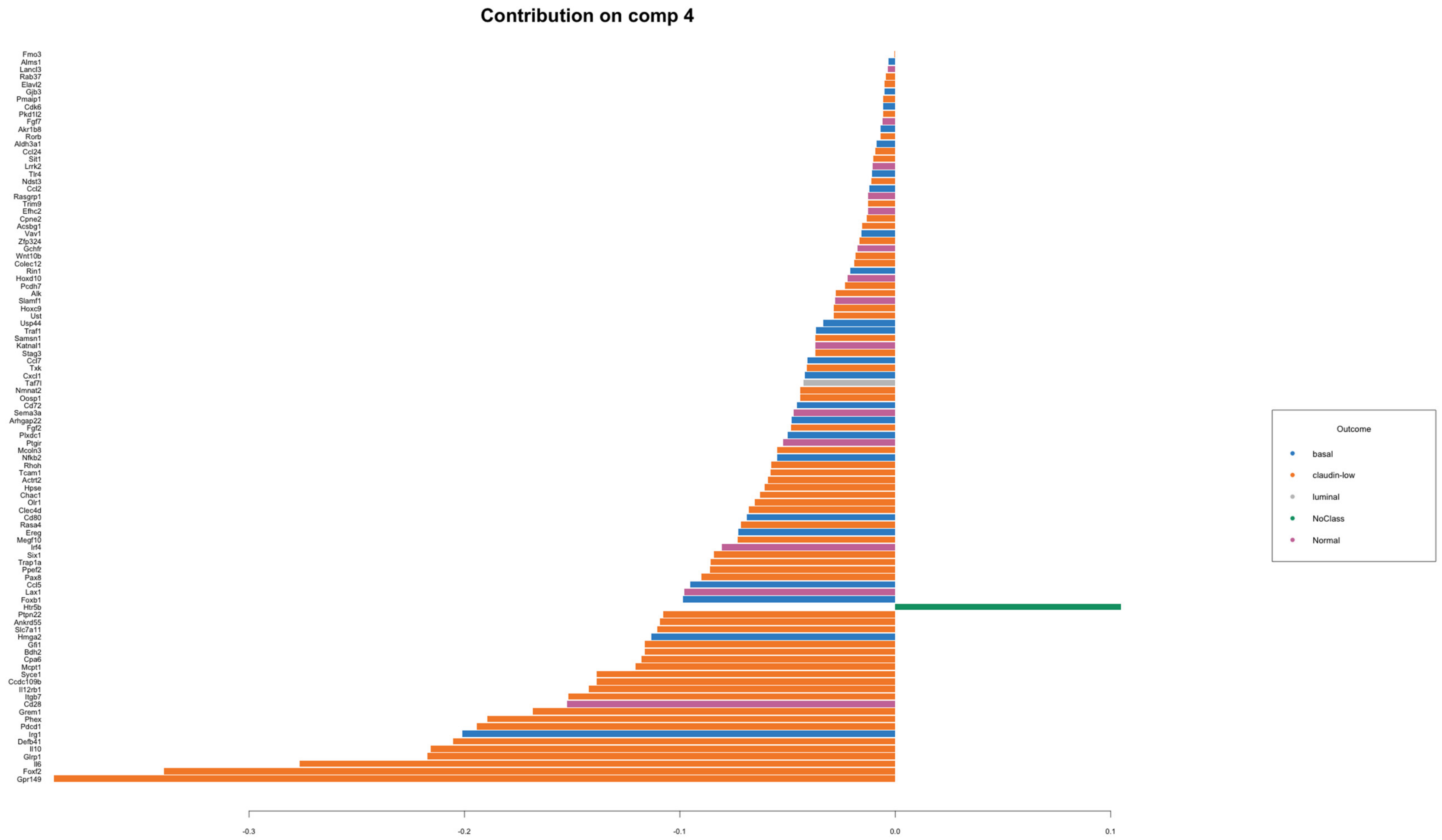

Figure S8. Classification analysis was performed using "mixOmics" R package 6.1.3. In the figure are reported the genes that discriminated among the three subtypes, luminal (n=8), basal (n=17) and claudin-low (n=2) in the component 4.
